# An unusual case of infective pneumocephalus: case report of pneumocephalus exacerbated by continuous positive airway pressure

**DOI:** 10.1186/s12873-018-0154-9

**Published:** 2018-01-18

**Authors:** Abdus Samad Ansari, Brittany B. Dennis, Dilip Shah, Winfred Baah

**Affiliations:** 1 Acute Medical Unit, Epsom and St. Helier University Hospital NHS Trust, London, England, UK; 2 St. George’s University of London, London, England, UK; 3 Department of Medicine, Korle Bu Teaching Hospital, P.O. Box KB77, Accra, Ghana

**Keywords:** Pneumocephalus, Osteomyelitis, Continuous positive airway pressure, Streptococcus salivarius, Clonus

## Abstract

**Background:**

Pneumocephalus, illustrated by air in the cranial vault is relatively infrequent and generally associated with neurosurgery, trauma, meningitis and barotrauma. However cases of spontaneous non-traumatic pneumocephalus remain rare. While the relationship between continuous positive airway pressure (CPAP) and atraumatic pneumocephalus has been previously reported, to our knowledge the rare presentation associated with sinus wall osteomyelitis has never been described. We summarize here the case of a 67-year-old woman’s acute presentation of *Streptococcus salvarius* infection after a sudden drop in her consciousness.

**Case presentation:**

The patient was brought to hospital by family reporting a one week history of sudden deterioration, cognitive decline, and lethargy. The patient presented with reduced arousal, cognitive function (Glasgow Coma Scale: 10, Abbreviated Mental Test Score:CS, 0 AMTS), and no history of trauma. Computed Tomography (CT) imaging was ordered and identified a significant pneumocephalus with no cranial defect. Further investigations acknowledged possible sinus or middle ear disease, which was highlighted by the discovery of *S. salivarius* by polymerase chain reaction (PCR) and potentially exacerbated by the use of nocturnal continuous positive airway pressure (CPAP). The patient made a complete recovery by eliminating likely causative factors and long term regimental antibiotics administration.

**Conclusion:**

This case highlights a rare neurological presentation of *S. salivarius* infection with a mixed aetiology of spontaneous pneumocephalus. This case features an atypical complication associated with CPAP use, and to our knowledge is the first case to be associated with sinus wall osteomyelitis. Recognition of the clinical features and risk factors for spontaneous pneumocephalus –while rare—serve to broaden our clinical index of suspicion when presented with patients experiencing neurological deficit. Information from this case may also aid in improving prevention, early diagnosis, and future management.

## Background

The presentation of pneumocephalus, illustrated by air in the cranial vault is relatively infrequent and generally associated with neurosurgery, trauma, and barotrauma [1]. It remains dependent on the drop of intracranial pressure associated with a defect within the dura or via a “ball-valve mechanism” linked to negative pressure with air entry [1].

However, cases of spontaneous non-traumatic pneumocephalus remain highly uncommon with previously reported causes secondary to malignancy, meningeal infection with gas forming organisms, and nasopharyngeal carcinoma [2]. Although the relationship with base of skull disease and pneumocephalus has been hypothesized, there is no literature describing such a case of osteomyelitis or the pathogensis of an associative organism. Due its pattern of colonization in conjunction with its proximity to the cranial vault, *Streptococcus salivarius* warrants consideration as an important potential source of infective pneumocephalus.

*S. salivarius* is known to be one of the first colonizers of the human oral cavity and gut after birth. Isolated strains of this pathogen found within the human pharynx have been shown to antagonistically work against respiratory pathogens, leding to its utility in many probiotics [3]. Cases of *S. salivarius* meningitis have been previously reported [4], however the majority of such cases occurred secondary to iatrogenic or traumatic causes. Patient’s susceptible for hematogenous transmission of *S. salivarius* from non-traumatic causes are those with adjacent infected areas. This could comprise a number of conditions including sinusitis, otitis media, and mastoiditis, of which *S. salivarius* is a common source [5]. Any irritation to the nasal passage, sinuses, or oropharyngeal tracts could serve as a mode of transmission for *S. salivarius.*

CPAP is an assisted breathing device which applies mild air pressure in a continuous fashion, used in patients able to breath spontenaously on their own and most commonly at night time for patients with obstructive sleep apnea (OSA). Case reports have noted CPAP as a source of air into the cerebrum among patients with previously unrecognized basal skull fractures [6]. However, to our knowledge no studies or case reports have ever described CPAP as a potential source of sponatenous pneumocephalus among patients with no evidence of skull fracture. We summarize here the case of a 67-year-old woman’s acute presentation of *S. salvarius* infection after a sudden drop in her consciousness. This case highlights a rare neurological presentation of *S. salivarius* infection with a mixed aetiology of spontaneous pneumocephalus. This case features an atypical complication associated with CPAP use, and to our knowledge is the first case to be associated with sinus wall osteomyelitis.

## Case presentation

**Fig. 1:**
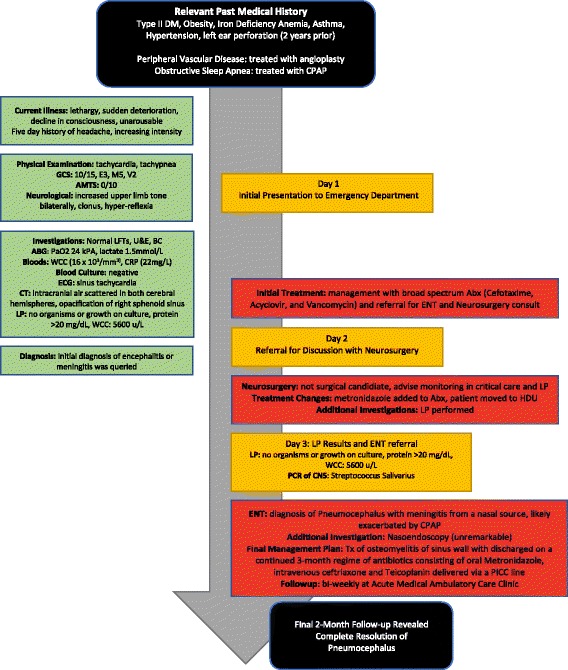
Case Timeline of Acute Presentation and Management. Liver Function Test (LFT), Urea and Electrolytes (U&E), Electrocardiogram (ECG), Blood Cultures (BC), Arterial Blood Gas (ABG), Computed Tomography (CT), White Cell Count (WCC), C-Reactive Protein (CRP), Glasgow Coma Scale (GCS), Abbreviated Mental Test Score (AMTS), Ear Nose and Throat (ENT), Lumbar Puncture (LP), High Dependency Unit (HDU), Antibiotics (Abx), Treatment (Tx)

A 67-year-old woman presented to the emergency department after being brought in by her family who described seven day symptoms of lethargy and marked deteroition. Collateral history revelaed the patient was housebound during this period, which was highly unusual for her. A dramatic decline in her consciousness leaving her unarousable at time of presentation pressed family to seek urgent medical attention. Retrospective history also revealed complaints of a five-day period of headaches steadily increasing in intensity. This was described as an ache radiating from her occiput to her forehead, with her unable to alleviate the pain with simple analgesia. She did not complain of visual disturbances, neck stiffness, or vomiting. Apart from a mild cough, runny nose, and slight nausea no other systemic symptoms were described. A timeline summarizing the events of this case is presented in Fig. 1; which includes a summary of the patient’s relevant medical comorbidities, presenting symptoms, investigations, as well as details of diagnosis and management.

The patient had a background of well controlled type II diabetes mellitus, asthma, hypertension, iron deficiency anemia (IDA) and peripheral vascular disease, treated with angioplasty three years prior to admission. The patient was also known to have a history of obstructive sleep apnoea, treated with nocturnal CPAP. Interestingly, the patient also reported a left ear perforation two years prior to admission.

On admission, initial observations noted the patient to be hypoxic, tacypneic and tachycardia. The patient’s GCS was recorded to be 10/15 E3, M5, V2. Neurological examination, although complicated by the reduced consciousness, noted increased tone bilaterally in her upper limbs, clonus and hyper-reflexia throughout. Arterial gas carried out on high flow oxygen (10 l) identified a PaO_2_ of 24 kPa, PaCO_2_ of 5.2 kPa, and mildly raised lactate (1.5 mmol/L). There were no signs of opthalmoplegia or facial asymmetry. The patient was nevertheless significantly disorientated in time, place and person; AMTS at time of admission was 0/10.

**Fig. 2:**
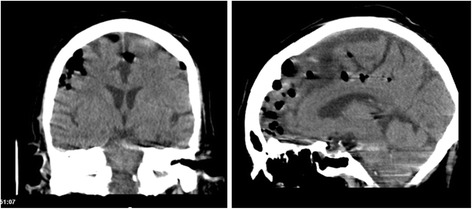
Coronal (left) and sagittal (right) CT imaging of cerebral hemispheres demonstrating multiple loculi of intracranial air

Laboratory results indicated raised inflammatory markers. White cell count: 16 × 10^3^/mm^3^, C-reactive protein: 22 mg/L, neutrophils: 13.8 × 10^3^/mm^3^ however renal and liver function tests were normal. ECG revealed the patient to be in sinus tachycardia. Due to the presentation, initial diagnoses of encephalitis and meningitis were queried. The patient was empirically started on Cefotaxime, Acyclovir, and Vancomycin in the emergency department. An urgent CT scan was arranged. Please refer to Fig. 2 for axial and sagittal CT images.

The CT imaging identified multiple loculi of intracranial air scattered in both cerebral hemispheres, which were not associated with an acute bleed or any midline shift. The images also display partial opacification of the right compartment of the sphenoid sinus as well as bilateral posterior ethmoid air cells. The imaging confirmed no evidence of fracture. Due to the absence of trauma or surgery, the appearances noted on the CT scan were reported to be secondary to sinus or middle ear disease.

The patient was discussed with our neurosurgical and neurology colleagues and deemed not to be for surgical intervention. In accordance with feedback from the neurosurgical team, the patient was stepped up to HDU management. This decision was based on the neurological indications for HDU admission. Metronidazole was subsequently added to treatment after advice by microbiology. Whilst in HDU the patient became acutely agitated and required sedation. Dexamethasone was started during stay in HDU. The patient did not receive ventilatory support in the form of CPAP, BIPAP, nor intubation whilst in HDU. Due to the severity of her intracranial symptoms, it was decided that the patient’s nocturnal CPAP management for sleep apnea was to be stopped during hospital stay.

Lumbar puncture revealed a turbid fluid with a white cell count (WCC) of 5600/ul (75% polymorphs 25% lymphocytes) and red blood cell count of 161/ul. The sample was found have protein >20.00 mg/dL and glucose of 3.9 mg/dL. The sample was positive for bilirubin and negative for both methaemoglobin and oxyhaemoglbin. Lab findings suggested no organisms were present in the sample in addition to no growth on extended culture.

*S. salivarius* was later isolated by PCR of the CSF. Blood cultures were negative. ENT review suggested a diagnosis of pneumocephalus with meningitis from a nasal source. The ENT assessment included a full history, clinical examination, nasoendoscopy, and careful review of CT imaging, where they noted no defects. The ENT team suggested the growth of *S. salivarius*, correlated with a diagnosis of sinusitis eroding back into the cranial vault. This was a diagnosis of exclusion after the growth of *S salivarius* was confirmed by PCR of the CNS fluid*,* suggesting a sinonasal source.

The patient’s recent history rhinorrhea in conjunction with the use of nocturnal CPAP and isolation of *S.Salvarius* on PCR led us to suspect this infection was due to osteomyelitis of the base of skull which may have precipitated a cranial defect, susceptible to the ‘one way valve mechanism’ often reported in cases of pneumocephalus. Thus without a defect noted on nasoendoscopy or CT imaging, it was believed the pneumocephalus was secondary to a cranial osteomyelitis - as suggested by the ENT and Microbiology team.

Outpatient treatment was started for likely osteomyelitis of the sinus wall. As the patient recovered, she was subsequently discharged on a continued 3-month regime of antibiotics consisting of oral Metronidazole, intravenous Ceftriaxone and Teicoplanin delivered via a PICC line. The patient’s CPAP was stopped for the duration of therapy for sinusitis and osteomyelitis. In addition a formal discussion was had with the patient about abstaining from CPAP during periods of sinusitis due to their previous susceptibility to infective pneumocephalus. Ensuring adherence and tolerability of the proposed management plan, the patient was reviewed bi-weekly in the hospital acute medical clinic. The ENT and neurosurgical team also carried out additional follow up appointments.

Repeat CT scan carried out two months after presentation revealed complete resolution of pneumocephalus. There was no intracranial haemorrhage, however mucosal thickening in the sphenoid and posterior ethmoid sinus was noted. No retro-orbital mass or sinus obstruction was found. Despite full resolve, the patient still complained of general lethargy and leg weakness at this point. There was however no change in personality or residual neurological deficit.

## Discussion

The term spontaneous or atraumatic pneumocephalus refers to conditions whereby air accumulates intracranially. Although the relationship with base of skull disease and pneumocephalus has been hypothesized, there is no literature reporting such a case of osteomyelitis related with *S. salivarius* and pneumocephalus with or without the use of CPAP.

Pneumocephalus was first reported in 1866 by Thomas during an autopsy of a trauma patient [7] and only linked as a complication of CPAP use in 1980 by Klopfenstein et al. whilst treating a patient with atelectasis [6]. In this case the patient was found to have an unrecognized basal skull fracture thus allowing the passage of air into the cerebrum. Current known causes of pneumocephalus include, cranio-facial trauma, air travel, trans-spehnoidal surgery, lumbar puncture, malignancy, meningeal infection with gas forming organisms and nasopharyngeal carcinoma [2].

A recent literature review by Pishbin et al. attempted to review all cases of spontaneous pneumocephalus reported [8]. They were able to identify 10 cases with a varying age between 10 and 74. Presenting symptoms remained relatively common, frequently linked to headache and nausea, however of these only one was complicated with CSF rhinorrhoea, precipitated by forceful sneezing. Treatment seemed to vary between surgery or conservative therapy dependant on aetiology and severity. Our patient’s retrospective history suggested a period of clear serous discharge nasally. Imaging was unable to find a cranial defect, thus initial suspicion suggested a meningeal infection with gas forming organisms, however the history of possible rhinorrhoea and use of nocturnal CPAP coupled with the growth of *S.Salvarius* would suggest this infection was due to osteomyelitis of the base of skull, precipitating a cranial defect susceptible to the ‘one way valve mechanism’ often reported in cases of Pneumocephalus. The rise in pressure caused by the CPAP machine resulted in forced air into the cranial cavity but due to the nature of the defect; it was not allowed to escape. In 2012 Wilson et al. reviewed literature identifying 65 cases of *S. salivarius* meningitis [4]. The vast majority of cases cited occurred secondary to iatrogenic or traumatic causes including, myelograma, lumbar punctures, neurosurgery, trauma and spontaneous dural defects. Of these they also reported one case of S.Salvarius meningitis associated with CSF rhinorrhea. This patient was also noted to have near complete opacification of the spehnoid sinus [4].

Symptoms of pneumocephalus are related to the amount of air that is within the cranial cavity. Although small amounts can be asymptomatic, larger amounts can have neurologically catastrophic outcomes. It is essential that any precipitating factors are eliminated immediately and adequate investigations are carried out to rule out infectious causes of disease. Our initial blood culture and lumbar puncture results did not identify any source of a bacterium. It was only after PCR analysis did we identify S. *Salvarius* to be the causative organism.

Common side effects of CPAP include trauma to the patient’s face such as nasal bridge or eye irritation, in combination with the negative effects of applying pressure and dry air on the airway [9]. Such effects include oropharyngeal dryness, epistaxis, rhinitis, and earache [9]. Rhinorrhea is an additional side effect occurring in up to 50% of patients with OSA using CPAP [10]. Due to the detrimental effects of the CPAP machine on both the naso and oropharyngeal tracts, it could be hypothesized that patients may be at increased risk for hematogenous spread of S. Salivarius, however to our knowledge this association has yet to be reported in the literature. Alternatively, CPAP has been linked to more serious adverse outcomes such as atraumatic pneumocelphalus. Acknowledging this has been reported in an exceptionally few number of patients, more attention must be given to the potential risk of pneumocelphalus assosciated with assisted breathing devices.

Further research is required to formally quantify the risk of infective pneumocephalus among patients using CPAP. Accordinlgy, headaches associated with cranial nerve deficits or a reduced GCS in those who use nocturnal CPAP should prompt clinicians to consider a diagnosis. Management and investigations between cases is often varied and it is essential these patients be managed under the guidance of ENT, Neurosurgical, Neurology and Acute Medical clinicians. Literature based on limited evidence and from animal models has empirically suggested a 6-week post-operative period after dural repair before the re-initiation of CPAP or BIPAP [6]. The purpose of this timeline, although varied between patients, involves carefully evaluating the risk of repeated pneumocephalus against that of apnea and hypoxia. A similar approach must be adopted in cases of atraumatic pneumocephalus. There remains a lack of formal guidance in the treatment of a non-traumatic pneumocephalus and thus further evaluation of best management and follow-up including re-initiation of CPAP is required to ensure optimal results and avoidance of reoccurrence.

## Conclusion

This case highlights a rare neurological presentation of *S. Salivarius* infection with a mixed aetiology of spontaneous pneumocephalus. This case features an atypical complication associated with CPAP use, and to our knowledge is the first case to be associated with sinus wall osteomyelitis. Recognition of the clinical features and risk factors for spontaneous pneumocephalus –while rare—serve to broaden our clinical index of suspscion when presented with patients experiencing neurological defecit. Information from this case will also aid in improving prevention, early diagnosis, and future management.

## Abbreviations

AMTS: Abbreviated mental test score
BIPAP: biphasic airway pressure
CPAP: Continuous positive airway pressure
CSF: Cerebral spinal fluid
CT: Computed tomography
ENT: Ear nose throat
GCS: Glasgow coma scale
PCR: polymerized chain reaction
PICC: Peripherally inserted central catheter
RBC: Red blood cell
